# A Recombinant 47-kDa Outer Membrane Protein Induces an Immune Response against *Orientia tsutsugamushi* Strain Boryong

**DOI:** 10.4269/ajtmh.15-0771

**Published:** 2017-06-05

**Authors:** Sangho Choi, Hang Jin Jeong, Kyu-Jam Hwang, Byoungchul Gill, Young Ran Ju, Yeong Seon Lee, Jeongmin Lee

**Affiliations:** 1 Division of Zoonoses, Center for Immunology and Pathology, National Institute of Health, Korea Centers for Disease Control and Prevention, Cheongju, Korea

## Abstract

We investigated the 47-kDa outer membrane protein (OMP), which is a periplasmic serine protease and an antigenic major surface protein of *Orientia tsutsugamushi*, as a vaccine candidate. We developed a conventional subunit vaccine expressing recombinant 47-kDa OMP (rec47) and a DNA vaccine (p47). In mouse immunization experiments, intranasal immunization with rec47 alone or with rec47 plus heat-labile enterotoxin B subunit from *Escherichia coli* or plus cholera toxin (CT) as adjuvants induced a higher amount of rec47-specific antibodies than intramuscular immunization with p47 alone or with p47 plus pBOOST2-samIRF7/3 (pB) as adjuvant. Moreover, the combination of rec47 and CT induced a strong cellular immune response to 47-kDa OMP, as demonstrated by a spleen cell proliferation assay, and also induced Th1- and Th2-type cytokine production, as demonstrated by a cytokine enzyme-linked immunosorbent assay. Intranasal immunization with rec47 plus CT was the most effective method for the induction of humoral and cell-mediated immune responses. Furthermore, relatively strong protection against homologous *O. tsutsugamushi* strain Boryong challenge was observed in mice immunized with rec47 plus CT. Therefore, 47-kDa OMP is an attractive candidate for developing a prophylactic vaccine against scrub typhus by *O. tsutsugamushi* infection.

## INTRODUCTION

Scrub typhus is an acute zoonotic disease caused by infection with the bacterium *Orientia tsutsugamushi*. The disease is transmitted by trombiculid mites (chiggers), and is characterized by fever, rash, eschar, pneumonitis, myocarditis, and disseminated intravascular coagulation.^1^^,^^2^ Scrub typhus is a critical public health concern in tropical areas in Asian countries, and it has been estimated that 1 billion people are at risk of infection, and 1 million cases are reported annually in the Asian-Pacific region.^3^ *Orientia tsutsugamushi* strains are antigenically diverse and divided into several serotypes.^4^ The endemic isolate most frequently found in Korea is the Boryong strain.^5^ In Korea, the disease incidence has increased due to environmental changes and increased outdoor activity leading to frequent exposure to chigger mites.^6^

Although scrub typhus can be treated with antibiotics such as doxycycline, tetracycline, and azithromycin, reinfection occurs frequently because of poor cross-reactive immunity and the short duration of protective immunity.^7^ Therefore, continuous efforts have been invested in the development of an effective prophylactic vaccine.^8^^–^^14^ In addition, vaccination would overcome difficulties in early clinical diagnosis and high mortality, and could limit the potential for developing antibiotic resistance.^15^^–^^18^ Several recombinant proteins have been tested for protection against homologous and/or heterologous strains in mice and nonhuman primates.^4^^,^^19^^–^^22^ However, no vaccine is available to date.

In the present study, we studied the feasibility of the 47-kDa outer membrane protein (OMP), a major OMP of *O. tsutsugamushi*, as a target for an effective vaccine. The protein belongs to the high-temperature requirement A (HtrA) family of serine proteases. The 47-kDa OMP is highly conserved in 25 highly disparate strains of *O. tsutsugamushi*,^23^ and contains both scrub typhus group-conserved and strain-specific B-cell epitopes.^24^^,^^25^ Because of its cross-reactive epitopes, it is a good vaccine candidate for broad protection against *Orientia* infection. In this study, we used intranasal and intramuscular immunization with recombinant 47-kDa OMP (rec47) and 47-kDa OMP-expressing DNA (p47) in mice. Immunization efficacy including humoral and cellular immune responses, as well as protection against a homologous challenge, were assessed.

## MATERIALS AND METHODS

### Ethics statement.

All animal care and experimental procedure conformed to the Korea National Institutes of Health (KNIH) guidelines (KNIH publication no. 11-1352173-000133-01, 2013) in accordance with National Guideline for the Care and Use of Laboratory Animals; the animal protocol used in the present study was reviewed and approved by the Institutional Animal Care and Use Committee of Korea Centers for Disease Control and Prevention (approval number: KCDC-040-14-2A).

### Bacterial strains and generation of recombinant plasmids.

The pathogenic *O. tsutsugamushi* Boryong strain, an endemic isolate from Korea, was used in this study. The pathogen was propagated in L929 cells (ATCC CLC-1) as described previously.^26^ The infected cells were incubated at 34°C in 5% CO_2_. At 3–4 days postinfection, infectivity was determined using an indirect immunofluorescence assay. Genomic DNA was extracted using the QIAamp genomic DNA kit (QIAGEN, Hilden, Germany). *Escherichia coli* TOP10 and BL21 DE3 strains (Invitrogen, Carlsbad, CA) were used for cloning and prokaryotic expression, respectively.

The primers OTBS1837-31-F (5′-GTGGATCCATGGTATTACCTCAACAAAAATC-3′) and OTBS1837-466-R (5′-GTCTCGAGTTACTTATTAATATTAGGTAAAGC-3′) were used for amplification of a truncated form (amino acids 31–466) of the 47-kDa OMP gene (GenBank accession number NC_009488.1). The polymerase chain reaction product was digested with *Bam*HI and *Xho*I, and cloned into the bacterial expression vector pRSET A (Invitrogen). The plasmid containing recombinant 47-kDa OMP (rec47) was transformed into *E. coli* BL21 (DE3) for overexpression. The bacterial culture conditions and the procedures for detection and purification of rec47 were as described previously.^27^

The nucleotides for the 47-kDa OMP DNA vaccine were synthesized with codon optimization for mammalian expression (Bioneer, Daejeon, Korea). The synthetic nucleotides were cloned into pVAX1 (Invitrogen) to generate the recombinant plasmid for mammalian expression p47. Protein expression was confirmed by transient expression of p47 in BHK-21 cells, using Lipofectamine 2000 Reagent (Invitrogen) according to the manufacturer's instructions.

### Immunization and challenge of mice.

Ninety-one 6-week-old female BALB/c mice (Charles River Laboratories, MA) were randomly divided into seven groups of 13 mice and used for immunization. In protein administration groups, mice were immunized intranasally with 10 μg of rec47 with or without 10 μg of heat-labile enterotoxin B subunit (LTB; Sigma Aldrich Co., Saint Louis, MO) from *E. coli* or 10 μg of cholera toxin (CT; List Biological Laboratories Inc., CA, USA) adjuvants. In DNA administration groups, mice were intramuscularly injected with 100 μg of p47 plasmid with or without 100 μg pBOOST2-samIRF7/3 (pB; InvivoGen, San Diego, CA). All immunizations were performed three times with 2-week intervals. At day 10 after each immunization, blood was collected from the mice by tail bleeding. Blood sera were recovered by centrifugation and stored at −20°C until further use. At day 10 after the third immunization, three mice of each group were euthanized and spleens were isolated for cell proliferation analysis.

For the protection study, 10 immunized mice of each group were challenged by intraperitoneal injection of 100 times the median lethal dose of the homologous *O. tsutsugamushi* Boryong strain (4.3 × 10^4^ colony-forming units) at 6 weeks after the third immunization. Mortalities were monitored daily for 2 weeks, and mice showing severe distress were euthanized for ethical reasons.

### Production of 47-kDa OMP-specific antibodies.

47-kDa OMP-specific immunoglobulins in the sera were titrated by indirect enzyme-linked immunosorbent assay (ELISA). Rec47 was used as an antigen, and the procedures were as described previously.^28^

### Determination of cytokine levels in serum.

A multiplex assay to measure the serum levels of cytokines including interferon gamma (IFN-γ), interleukin (IL)-2, IL-4, IL-5, IL-6, IL-10, and IL-12 (p70) in immunized mice was performed with the Luminex 200 System (Merck Millipore, Darmstadt, Germany) according to the manufacturer's instructions.

### Determination of cytokine release in spleen cells.

For the measurement of cytokines including IFN-γ, tumor necrosis factor (TNF)-α, IL-2, IL-5, IL-6, and IL-10 released from the immune cells, spleen cells of immunized mice were isolated and seeded onto six-well plates (1 × 10^7^ cells/well). For restimulation, rec47 was added to each well (10 μg/mL), and the plate was incubated at 37°C in 5% CO_2_ for 24 hours. Cytokines were measured using the Duoset ELISA Development kit (R&D Systems Inc., Minneapolis, MN) according to the manufacturer's instructions.

### Spleen cell proliferation assay.

For the measurement of the cell-mediated immune response specific to 47-kDa OMP, a cell proliferation assay was performed in vitro. Spleen cells from the spleen of immunized mice were isolated and seeded onto 96-well plates (1 × 10^6^ cells/well). For restimulation, rec47 (1 μg/mL) was added to each well, and the plate was incubated at 37°C in 5% CO_2_ for 72 hours. Cell proliferation was assessed at 24, 48, and 72 hours using the EZ-CYTOX assay kit (Daeil Laboratory Service, Seoul, Korea) according to the manufacturer's instructions.

### Statistical analysis.

All assays were repeated at least three times. The data are expressed as the mean ± standard deviation. The statistical significance of differences between multiple groups was determined by one-way analysis of variance. Student's two-tailed *t* test was used to compare the means of the experimental and control groups. *P* values of < 0.05 or < 0.01 were considered significant.

## RESULTS

### Production of the rec47 and p47 vaccines.

The rec47 subunit vaccine with an N-terminal 6-histidine tag was produced as an insoluble protein expressed in *E. coli* BL21 (Supplemental Figure 1A). The expression yield of rec47 was approximately 30% of total cellular protein (data not shown). To evaluate antigenic reactivity, purified rec47 was subjected to sodium dodecyl sulfate polyacrylamide gel electrophoresis (SDS-PAGE) (Supplemental Figure 1B) and western blotting (Supplemental Figure 1C). The rec47 was clearly detected using serum from a scrub typhus patient showing IgG titer > 1:2,048.

The sequence of the codon-optimized DNA vaccine was confirmed by sequencing, and inserted into pVAX1 to generate p47, which contained an IL-2 signal peptide and a 6-histidine tag for secretion and detection, respectively (Supplemental Figure 2A). Expression of the recombinant protein was detected in BHK-21 cell lysates within 2 days after transfection (Supplemental Figure 2B).

### Humoral immune responses in mice.

**Figure 1.**
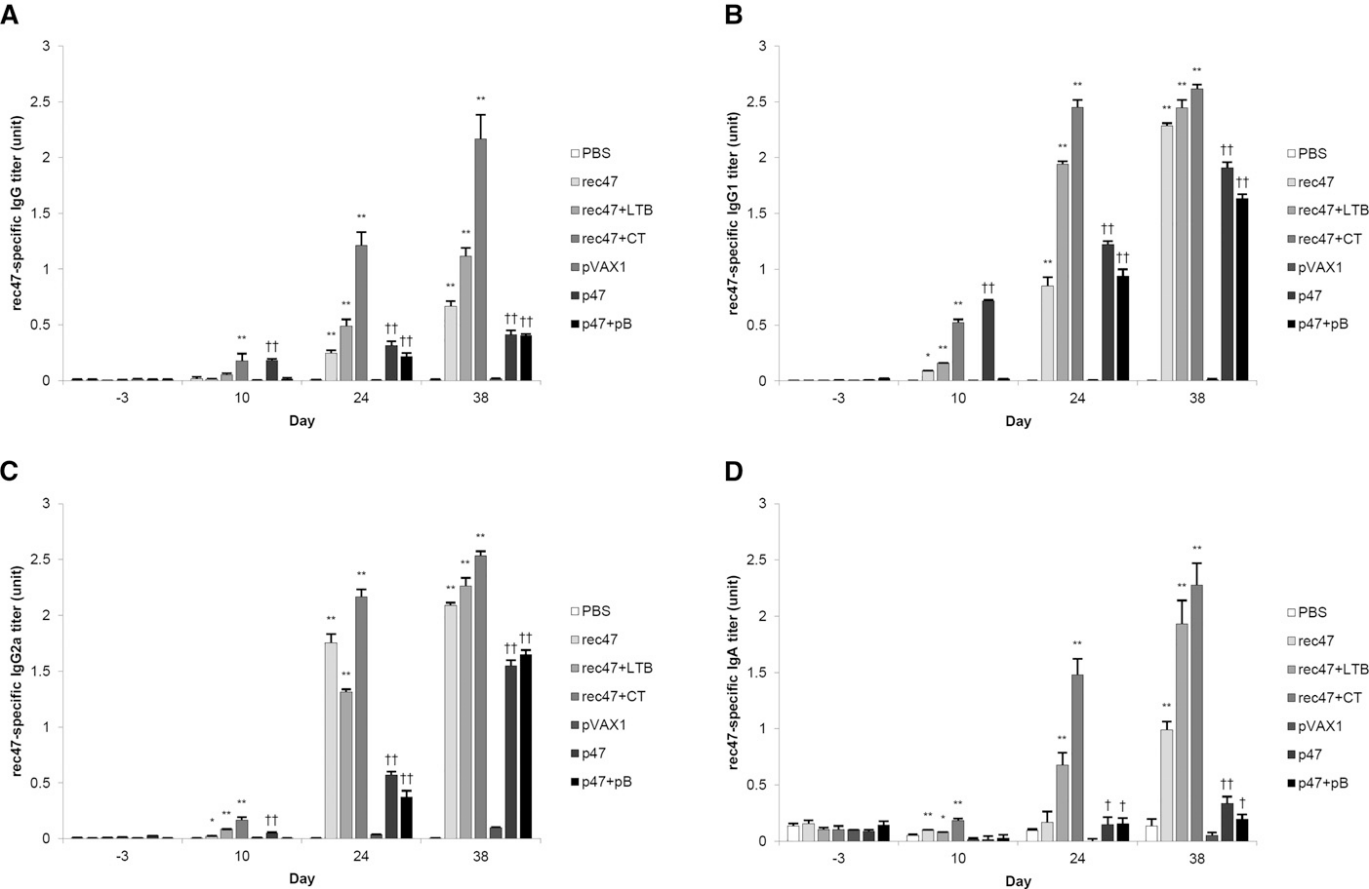
Humoral immune response upon the different treatments, as measured in sera from immunized mice. 47-kDa outer membrane protein–specific immunoglobulins including total (**A**) IgG, (**B**) IgG1, (**C**) IgG2a, and (**D**) IgA were measured by indirect enzyme-linked immunosorbent assay after every immunization. Statistically significant differences between the PBS control group and each recombinant protein immunization group are indicated with * (*P* < 0.05) or ** (*P* < 0.01). Statistically significant differences between the pVAX1 control group and each DNA vaccination group are indicated with † (*P* < 0.05) or †† (*P* < 0.01).

The schedule for immunization, bleeding, and specimen collection is presented in Supplemental Figure 3. The titers of the anti-47-kDa OMP antibody were measured by indirect ELISA. The serum IgG levels in all experimental groups significantly increased after the second immunization (day 24), compared with the negative controls, PBS and pVAX1 control groups (*P* < 0.01), with high variation between the groups. The levels of IgG further increased after the third immunization (day 38). All rec47-immunized groups, with or without adjuvant, showed higher IgG titers than the p47-immunized groups (Figure 1A

), with rec47 plus CT displaying the highest titer at day 38. IgG subclass analysis revealed that IgG1 and IgG2a significantly increased in all experimental groups after the second immunization, and further increased after the third immunization (*P* < 0.01). IgG1 and IgG2a titers were present relatively highly in total IgG in the p47-immunized groups, as demonstrated by the results in Figure 1A–C. Immunization with rec47 induced significantly higher levels of IgA than immunization with p47 (Figure 1D) at day 38.

### Cellular immune responses in mice.

**Figure 2.**
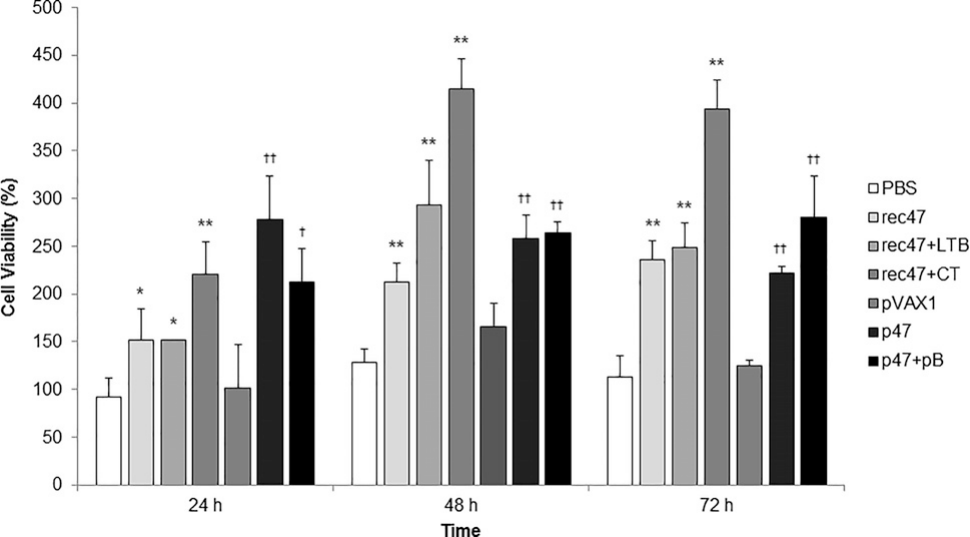
Spleen cell proliferation assay to measure cell-mediated immunization. The isolated spleen cells were restimulated with 1 μg of rec47. Cell viability was measured as the percentage of viable cells at 24, 48, and 72 hours after restimulation. Statistically significant differences between the PBS control group and each recombinant protein immunization group are indicated with * (*P* < 0.05) or ** (*P* < 0.01). Statistically significant differences between the pVAX1 control group and each DNA vaccination group are indicated with † (*P* < 0.05) or †† (*P* < 0.01).

Spleen cells obtained from the immunized mice were restimulated for 24, 48, and 72 hours with rec47, and cell proliferation was recorded (Figure 2

). The fraction of viable cells was higher for the p47-immunized groups than for the rec47-immunized groups after a 24-hour stimulation. All immunized groups showed significantly higher levels of cell proliferation than the control groups after a 48-hour stimulation (*P* < 0.01). The highest viability was observed for cells from mice immunized with rec47 plus CT at 48 and 72 hours after restimulation.

### Cytokine levels in serum.

**Figure 3.**
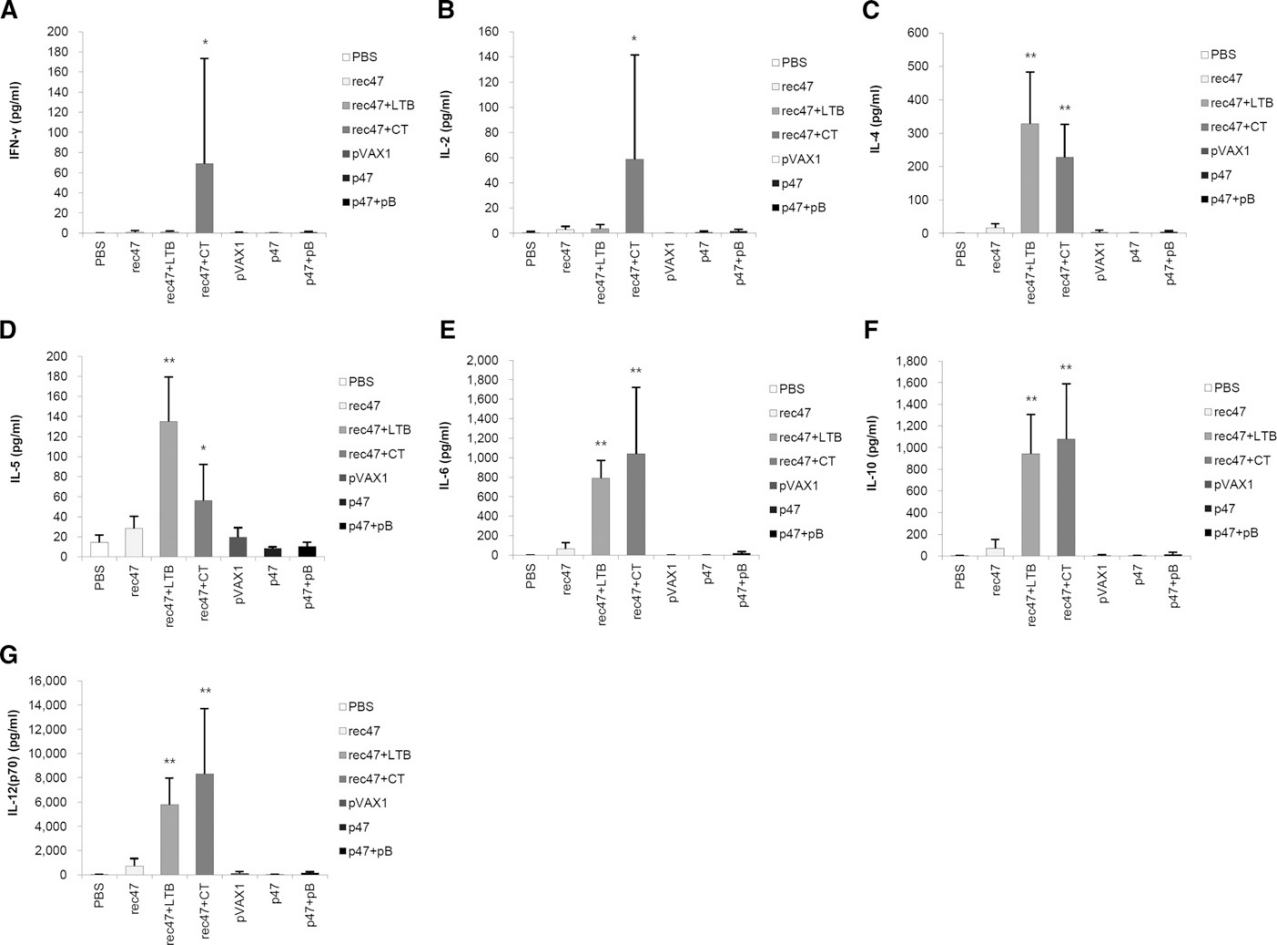
Cytokine release in immunized mice sera. After the third immunization, the concentration of distinct Th1- and Th2-type cytokines in mouse serum was measured by using a multiplex assay. (**A**) interferon gamma (IFN-γ), (**B**) interleukin (IL)-2, (**C**) IL-4, (**D**) IL-5, (**E**) IL-6, (**F**) IL-10, and (**G**) IL-12 (p70). Statistically significant differences between the PBS control group and each recombinant protein immunization group are indicated with * (*P* < 0.05) or ** (*P* < 0.01). Statistically significant differences between the pVAX1 control group and each DNA vaccination group are indicated with † (*P* < 0.05) or †† (*P* < 0.01).

Th1- and Th2-type cytokine levels in mice sera were measured after the third immunization. An increase in both Th1- and Th2-type cytokines was observed in the groups immunized with rec47 compared with the control group, especially for rec47 in combination with LTB or CT adjuvant (Figure 3

). Significantly higher levels of IFN-γ, IL-2, and IL-12 (p70) were observed for the rec47 plus CT group. Cytokine levels in the sera from mice immunized with p47 or with p47 plus pB were much lower and not significantly different from the control group.

### Cytokine secretion from splenocytes.

**Figure 4.**
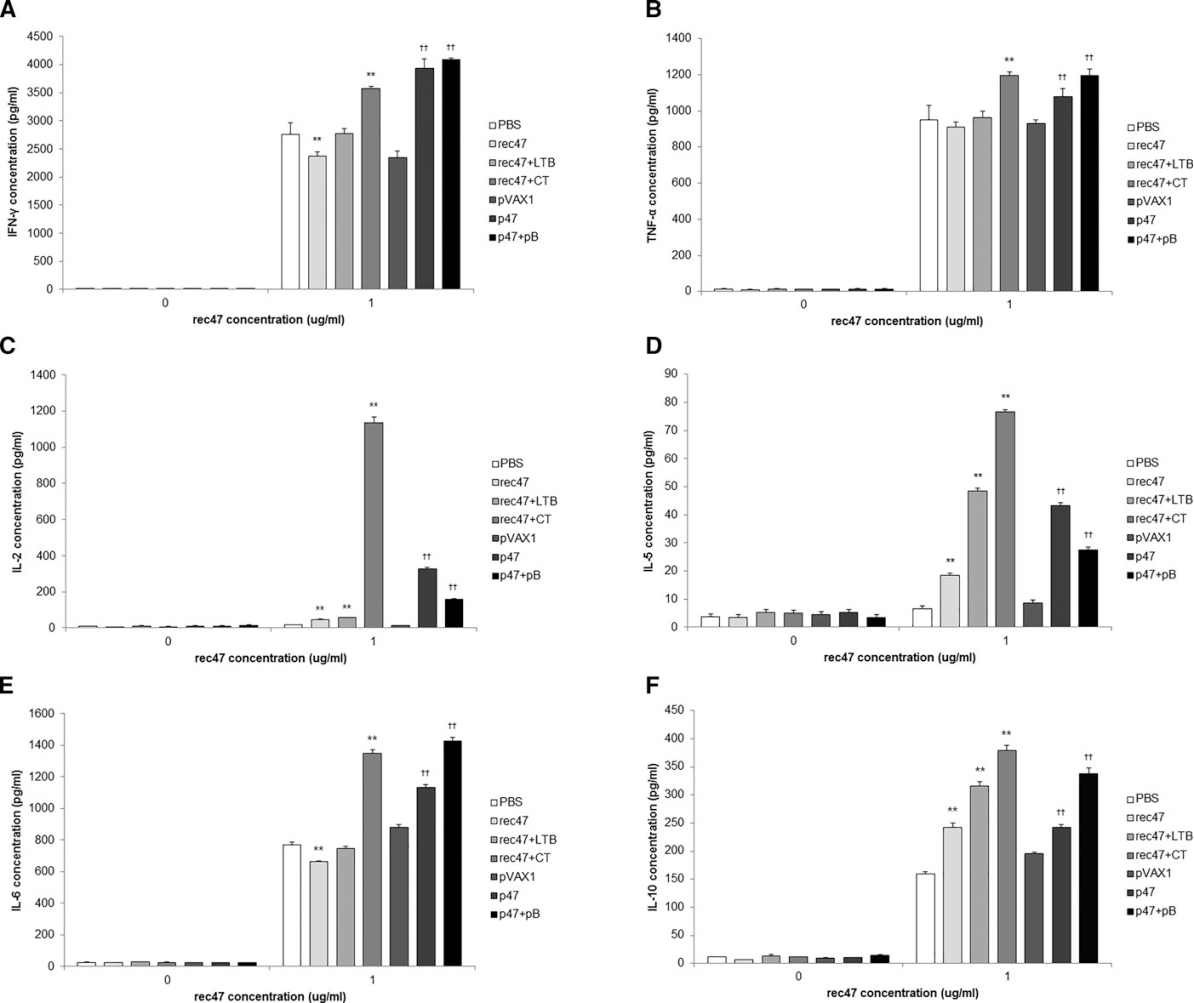
Cytokine release from spleen cells isolated from immunized mice. The isolated spleen cells were restimulated with 1 μg of rec47. Cytokines were measured at 24 hours after restimulation using an enzyme-linked immunosorbent assay. (**A**) interferon gamma (IFN-γ), (**B**) tumor necrosis factor (TNF)-α, (**C**) interleukin (IL)-2, (**D**) IL-5, (**E**) IL-6, and (**F**) IL-10. Statistically significant differences between the PBS control group and each recombinant protein immunization group are indicated with * (*P* < 0.05) or ** (*P* < 0.01). Statistically significant differences between the pVAX1 control group and each DNA vaccination group are indicated with † (*P* < 0.05) or †† (*P* < 0.01).

We measured the secretion of Th1-type cytokines including IFN-γ, TNF-α, and IL-2, and Th2-type cytokines including IL-6, IL-5, and IL-10, by spleen cells from immunized mice (Figure 4

). The Th1-type cytokine level in all protein vaccination groups except rec47 plus CT was equal to or lower than that in the control group. In contrast, all the p47-immunized groups, with or without adjuvant, showed significantly higher levels of Th1-type cytokines than the control group (*P* < 0.01). Interestingly, although the IFN-γ level in the group immunized with rec47 plus CT was lower than that in the DNA-immunized groups, IL-2, IL-5, and IL-10 levels in the group immunized with rec47 plus CT were significantly higher (*P* < 0.01) than in other groups, with the most remarkable difference noted for IL-2. Although it belongs to the Th2-type cytokines, IL-6 showed an induction pattern similar to that of the Th1-type cytokines IFN-γ and TNF-α. While the rec47 adjuvants had a positive effect on the cytokine secretion, the DNA adjuvant pB showed a positive effect on IFN-γ, TNF-α, IL-6, and IL-10, whereas it negatively affected IL-2 and IL-5 secretion.

### Protective immunity conferred by 47-kDa OMP in mice.

**Figure 5.**
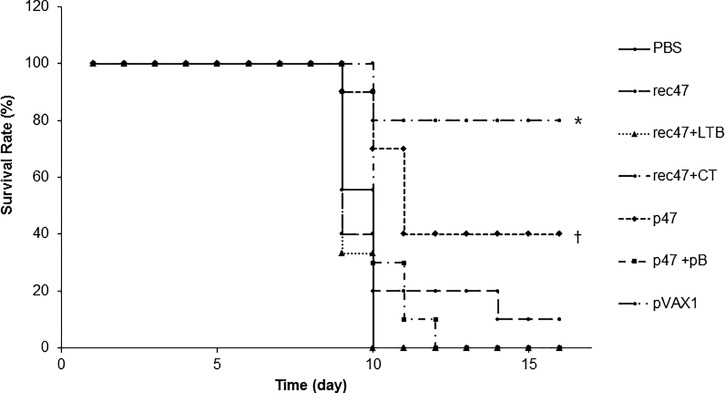
Evaluation of the protection against homologous challenge in immunized mice. *Orientia tsutsugamushi* strain Boryong was injected intraperitoneally into mice immunized with rec47 alone or rec47 plus LTB/CT and into mice immunized with p47 alone or p47 and pB, and their respective control treatments. The mortality was monitored daily for 2 weeks. The survival rate was calculated as the ratio of the number of living mice to the total number of challenged mice in a group. Statistically significant differences between the PBS control group and each recombinant protein immunization group are indicated with * (*P* < 0.05). Statistically significant differences between the pVAX1 control group and each DNA vaccination group are indicated with † (*P* < 0.05).

The mice showed clinical symptoms of shiver, piloerection, loss of body weight, and decease in activity at day 7–9 after challenge, and the most cases of death were observed at day 9–12. After day 15, the survived mice were completely recovered or any defect or abnormal feature was not observed. The highest protection against homologous challenge with *O. tsutsugamushi* strain Boryong was achieved in the rec47 plus CT-immunized mice with a survival rate of 80% 15 days after challenge (*P* < 0.05), followed by the mice immunized with p47 alone (40% survival; *P* < 0.05) and rec47 alone (10% survival; *P* > 0.05) (Figure 5

). Mice immunized with rec47 plus LTB or p47 plus pB, and control mice (PBS or pVAX1), did not survive.

## DISCUSSION

To date, research efforts to develop a scrub typhus vaccine have been focused on the elucidation of immunogenic antigens and their roles in inducing both humoral and cellular immunity in response to *O. tsutsugamushi* infection. In this study, we investigated the feasibility of the 47-kDa OMP as a vaccine candidate.

The 47-kDa OMP contains a region that exhibits a high sequence homology with human serine protease HtrA1, thus it was suggested that multiple immunizations of p47 may induce autoimmune responses.^23^ However, previous studies showed that immunization with 47-kDa OMP derivatives elicited a strong immune response: Balb/c mice immunized with recombinant 47-kDa OMP or a 40-kDa fragment showed increased spleen cell proliferation and IgG production.^29^ A DNA vaccine based on the *O. tsutsugamushi* Karp strain 47-kDa protein gene effectively protected CD-1 outbred mice against homologous challenge.^30^ In a nonhuman primates model using 47-kDa OMP of Karp strain, a DNA plasmid vaccine (pKarp47) showed induction of sterile immunity against high-dose homologous intradermal challenge of *O. tsutsugamushi*; however, a virus-vectored vaccine (Kp47-VRP) itself was not effective for protection and elimination of bacteremia.^31^

We used the N-terminal truncated rec47 based on the *O. tsutsugamushi* Boryong strain 47-kDa protein without any carrier as an antigen to induce mucosal as well as systemic immunity. Intranasal injection was selected for optimal administration of rec47 based on previous (unpublished, by Hyuk Chu) findings. LTB and CT were used as mucosal adjuvants, because they are known to enhance mucosal immune responses that had been proven a problem to be solved for intranasally administered vaccinations.^32^^–^^36^

Immunization with rec47 alone or with rec47 plus LTB or CT induced higher humoral immunoglobulin production than DNA immunization with p47 or with p47 plus pB at days 24 and 38. However, the spleen cell proliferation assay revealed no significant difference in cell viability after restimulation between groups immunized with rec47 and groups immunized with p47, except for rec47 plus CT. These results indicate that, although the protein vaccine induced a stronger humoral response, it did not provide more effective protection against homologous infection, unless it was used in combination with CT.

The addition of LTB or CT significantly increased rec47-specific IgG and IgA production upon the second immunization. In particular, CT had the largest effect on the humoral immune response to rec47. Moreover, rec47 plus CT induced the strongest Th1-type immune response upon restimulation in mice and spleen cells. In addition, rec47 with CT immunization induced high levels of Th2-type cytokines associated with the humoral immune response in mice and spleen cells. Therefore, rec47 plus CT was the most effective in inducing humoral as well as cellular immune response.

The 47-kDa OMP DNA vaccine that we used in this study was codon optimized for expression in mice. We assessed the additional effect of the pBOOST2-samIRF7/3 plasmid, which has been developed as a genetic adjuvant for DNA vaccines to potentiate the immune response to a specific antigen. This plasmid encodes a chimeric protein from the interferon regulatory factor family, which can enhance both the Th1 and Th2 responses in T cells, leading to the activation of cytotoxic T cells and/or the production of antibodies.^37^^,^^38^ In our study, this adjuvant showed no significant positive effect on antibody production and spleen cell proliferation. However, cytokine secretion patterns in splenocytes from mice immunized with p47 alone or with p47 plus pB differed after restimulation; while IL-2 and IL-5 levels were reduced, IL-6 and IL-10 production were stimulated by addition of the adjuvant.

Evaluation of the immunological protection against homologous challenge by the vaccines showed that immunization with rec47 plus CT elicited the strongest protection against scrub typhus compared with other immunization methods. Cellular immune responses to rickettsial infections have been shown to coincide with the development of protection from secondary infection in mice, and the resistance was associated with an increase in lymphocyte proliferation.^39^ In our study, high survival rates upon immunization with rec47 plus CT were associated with high IFN-γ and IL-2 levels. IFN-γ and IL-2 are both produced by CD4^+^ T (Th1-type) cells, and IFN-γ has been shown to inhibit rickettsial growth in vitro and is believed to play an important role in resistance to rickettsial infection in vivo.^4^^,^^40^^–^^43^ Several previous reports have suggested that protective immunity to scrub typhus is based on the development of cell-mediated rather than humoral immunity,^44^ which has been demonstrated by the induction of resistance by alloserum containing immune T cells,^45^ by the development of delayed-type hypersensitivity (DTH),^46^ and by in vitro cytokine secretion by T cells in response to *O. tsutsugamushi* antigens.^25^^,^^42^^,^^43^^,^^47^ Th1 cells were shown to be responsible for the DTH response, which correlated with resistance to lethal-dose challenge with *O. tsutsugamushi* in mice.^39^^,^^46^ However, other studies have suggested that the humoral immune response plays a prominent role in protective immunity by inhibiting the attachment and/or penetration of the pathogen,^48^ and the group- and strain-specific epitopes of the 47-kDa OMP were recognized by mouse T cells in a previous study.^25^ In this study, we did not find a clear correlation between the antibody titers and protection, and we observed discordance between cell-mediated immunity and protection.

The survival rate of mice immunized with the DNA vaccine p47 was significantly lower than that of mice treated with rec47 plus CT. However, DNA vaccines have many advantages over conventional vaccines such as the induction of a strong cell-mediated immune response, the elimination of safety concerns associated with live vaccine organisms, the ease of development, and their high productivity, stability, and economic feasibility.^49^^–^^51^ The feasibility of p47 as a DNA vaccine may be significantly improved by several approaches, including enhancement of the expression and secretion of the encoded protein, co-immunization with appropriate adjuvants, cloning the gene into dendritic cell-attracting vectors to enhance 47-kDa OMP presentation, and using liposomes for a more efficient delivery system.^22^ Immunization with p47 plus pB induced a similar level of antibody production and cell proliferation as immunization with p47 alone, and induced a higher level of INF-γ and TNF-α. However, the survival rate upon homologous challenge was 0%, which was lower than that of immunization with p47 alone. Therefore, pB had a limited adjuvant effect and even inhibited the protection effect of p47 against *O. tsutsugamushi* infection.

In conclusion, we showed that the recombinant 47-kDa OMP subunit vaccine and the 47-kDa OMP-expressing DNA vaccine induced cell-mediated immune responses and antibody production specific to 47-kDa OMP. Relatively strong protection against homologous challenge was observed in mice immunized with rec47 plus CT. Therefore, 47-kDa OMP is an attractive candidate for developing a prophylactic vaccine against scrub typhus by *O. tsutsugamushi* infection. Although the 47-kDa OMP alone may have a low efficacy in inducing heterologous protection, a multisubunit or multivalent vaccination, combined with other effective adjuvant(s), may be a promising approach to create full protection against scrub typhus by *O. tsutsugamushi* infection.

## Supplementary Material

Supplemental Figure.
