# Supplementary material for: A Recombinant 47-kDa Outer Membrane Protein Induces an Immune Response against Orientia tsutsugamushi Strain Boryong
Source: Am J Trop Med Hyg. 2017 Jun 5;97(1):30–7. doi: 10.4269/ajtmh.15-0771 (PMC5508880; doi:10.4269/ajtmh.15-0771)
Supplement: Supplementary file 1 [file tpmd150771.SD1.pdf]

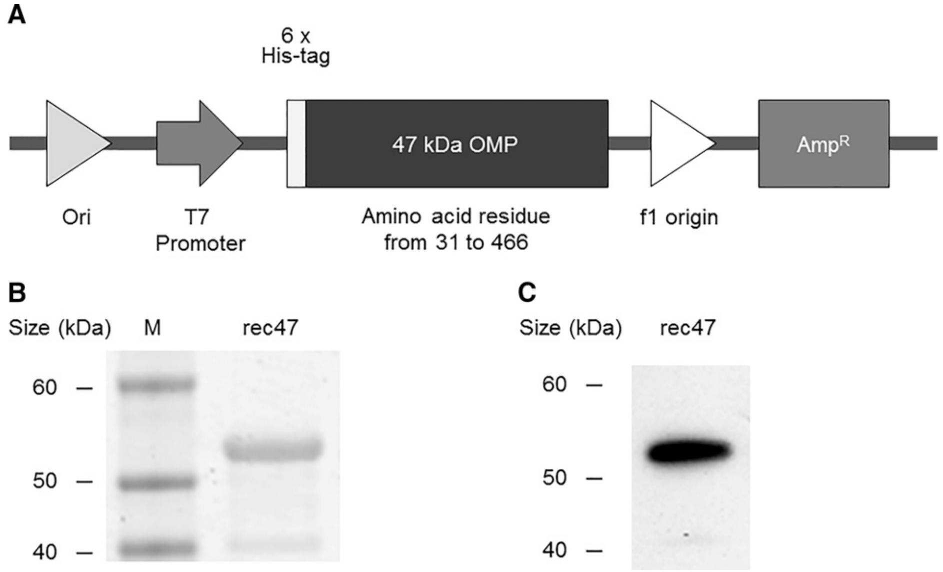

Supplemental Figure 1. Construction and sodium dodecyl sulfate polyacrylamide gel electrophoresis (SDS-PAGE) and western blot analyses of the recombinant 47-kDa outer membrane protein (OMP) (rec47). (**A**) Schematic diagram of the expression vector for rec47 production in *Escherichia coli*. A truncated form of the recombinant 47-kDa OMP (amino acids 31–466) was produced. (**B**) SDS-PAGE analysis of the purified rec47. (**C**) Western blot analysis of rec47 using anti-sera from scrub typhus patients confirmed by immunofluorescence assay (IgG titer > 1:2,048).

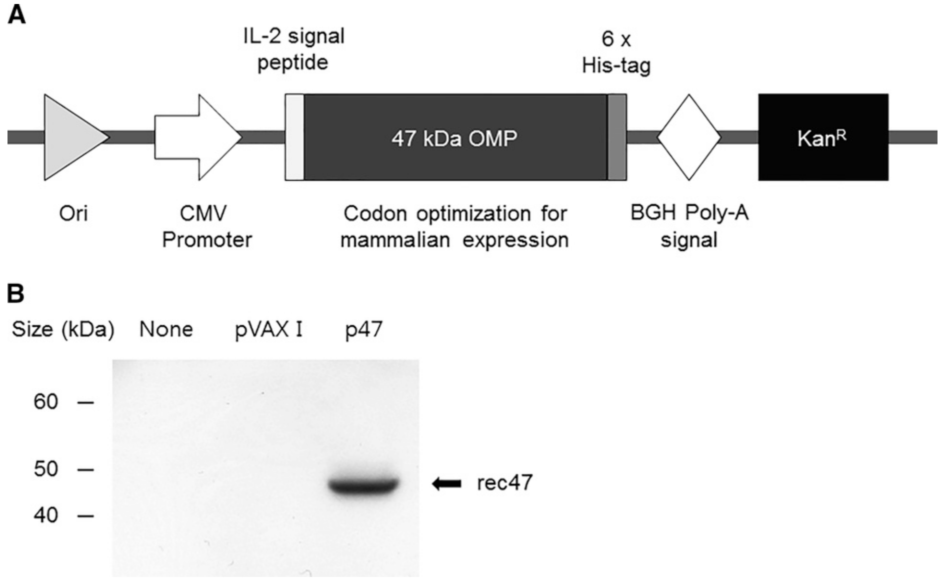

Supplemental Figure 2. Construction and mammalian expression of the 47-kDa outer membrane protein (OMP) DNA vaccine. (**A**) Schematic diagram of the expression vector p47 with codon optimization for mammalian expression. (**B**) Transient expression of the recombinant 47-kDa OMP in hamster kidney fibroblast (BHK-21) cells after transfection with the p47 plasmid. The expression of 47-kDa OMP was detected by using anti-His-tag antibody.

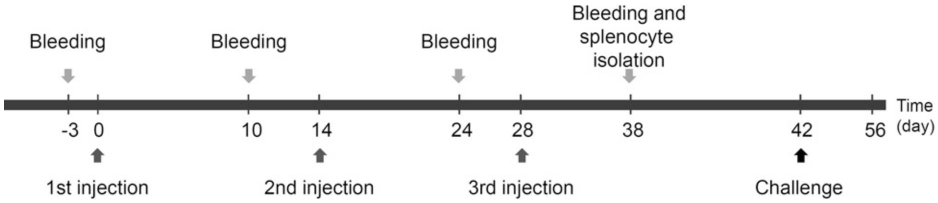

Supplementary Figure 3. Time line of the mouse immunization procedure. Ninety one 6-week-old female mice were randomly divided into seven groups according to the treatment they were subjected to: negative control treatment with PBS, rec47 alone, rec47 plus LTB, rec47 plus CT, p47 alone, and p47 plus pB. The immunizations were performed by intranasal administration of rec47 (10 μg) alone or of rec47 plus LTB/CT (1 μg), or by intramuscular injection of p47 plasmid (100 μg) alone or of p47 plus pB (100 μg).
